# “Social Life” of Senescent Cells: What Is SASP and Why Study It?

**Published:** 2018

**Authors:** A. V. Borodkina, P. I. Deryabin, A. A. Giukova, N. N. Nikolsky

**Affiliations:** Institute of Cytology, Russian Academy of Sciences, Tikhoretsky Ave. 4, St. Petersburg, 194064, Russia

**Keywords:** antagonistic pleiotropy, cellular senescence, immune clearance, senescence-associated secretory phenotype, stem cells, tumor suppression, tumorigenesis

## Abstract

Cellular senescence was first described as a failure of normal human cells to divide indefinitely in culture. Until recently, the emphasis in the study of cell senescence has been focused on the accompanying intracellular processes. The focus of the attention has been on the irreversible growth arrest and two important physiological functions that rely on it: suppression of carcinogenesis due to the proliferation loss of damaged cells, and the acceleration of organism aging due to the deterioration of the tissue repair mechanism with age. However, the advances of the past years have revealed that senescent cells can impact the surrounding tissue microenvironment, and, thus, that the main consequences of senescence are not solely mediated by intracellular alterations. Recent studies have provided evidence that a pool of molecules secreted by senescent cells, including cytokines, chemokines, proteases and growth factors, termed the senescence-associated secretory phenotype (SASP), via autocrine/paracrine pathways can affect neighboring cells. Today it is clear that SASP functionally links cell senescence to various biological processes, such as tissue regeneration and remodeling, embryonic development, inflammation, and tumorigenesis. The present article aims to describe the “social” life of senescent cells: basically, SASP constitution, molecular mechanisms of its regulation, and its functional role.

## INTRODUCTION

**Fig. 1:**
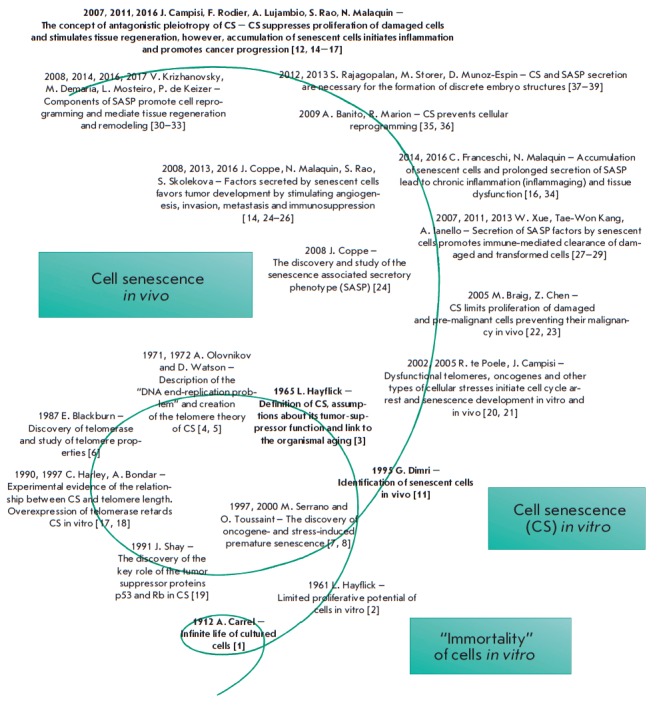
The timeline of cellular senescence research

The history of cellular senescence (CS) studies can be viewed within the framework of the well-known dialectic law of “negation of the negation,” which represents a process of development as a spiral (*Fig. 1*). The first turn of this imaginary spiral dates back to more than 100 years ago and reflects a view that had remained the prevailing one in science for a long time: aging is a phenomenon unique to organisms and can be avoided in cell culture. The basic proof of this hypothesis was gathered and published in the work of Nobel Laureate A. Carrel [1]. In his experiments, Carrel demonstrated the feasibility of endless proliferation of cells in culture, given adequate conditions, sufficient quantities of nutrients and, as he himself put it, “due diligence.” The paradigm shift and the transition to a new turn in the spiral occurred almost 50 years later, thanks to the work of L. Hayflick, who established the existence of a limit in the division of normal human fibroblasts* in vitro *[2]. Later, this limit was named the Hayflick limit, and the author himself interpreted his findings as a manifestation of human aging at the cellular level [3]. The next important stage in the study of cellular senescence dates back to the early 1970s, when independently of each other A. Olovnikov and D. Watson described the issue of terminal DNA underreplication [4, 5]. According to this hypothesis, the 5’-terminal daughter DNA chain is shortened with each cell division, which ultimately leads to the Hayflick limit. This discovery led to the elucidation of the telomere theory, according to which telomere shortening is what mediates replicative senescence [4]. Shortly afterwards, the structure of telomeres was elucidated and their properties were investigated [6]. Approximately at the same time, other authors began publishing papers which indicated that there is another type of CS that is independent of telomere length [7, 8]. This type of senescence was called ‘premature senescence,’ since its signs manifested themselves in cells during early passages, long before the onset of replicative senescence. Various stress factors and overexpression of oncogenes are considered to be the main inducers of premature senescence [7-10].

Despite the progress achieved in the study of the mechanisms of CS, for a long time the relationship between cellular and organismal aging remained hypothetical. The experimental evidence for the existence of senescent cells in human tissue samples was obtained only in 1995 [11]. By linking the processes taking place* in vivo *and *in vitro*, these observations drew a line under the previous turns of the spiral and serve as the point of origin for the next phase, which continues up to this day. Previously, manifestations of CS at the organismal level had been considered as something unidirectional, associated exclusively with age and age-related diseases. Today, the effects of CS are described using the concept of antagonistic pleiotropy, implying a role in the most diverse and sometimes opposite processes, such as repair, regeneration, tissue remodeling, embryogenesis, inflammation, tumor suppression and tumorigenesis [12-16].

## PHENOMENOLOGY OF CELLULAR SENESCENCE

Before delving into the heart of this review, which is devoted to the changes that accompany CS and its role in various biological processes, one needs first to understand the essence of this phenomenon. From a mechanistic point of view, the term CS implies an irreversible loss of the proliferative potential of metabolically active cells, which is caused by irreparable DNA damage [40]. If CS is considered at the organismal level, it becomes obvious that preventing the proliferation of cells that are damaged due to their senescence upholds tissue homeostasis. The generally accepted view of the moment that logically follows from the statements above is that senescence is exclusively characteristic of proliferating cells.

During ontogenesis, cell proliferation begins from the moment of the first fragmentation of the zygote. The blastomeres formed as a result of mitotic divisions and subsequent embryonic stem cells (ESCs) are known to possess an unlimited replicative potential. At the molecular level, ESCs lack of replicative senescence is mediated by telomerase activity, which compensates for the shortening of telomeres in each cell division [41, 42]. It is important that these cells also do not exhibit premature senescence: in case of irreparable damage, ESCs are eliminated from the population by apoptosis, which is necessary to preserve the stability of the genome [43]. Due to unrestricted proliferation and their ability to differentiate, ESCs give rise to all types of cells in an adult organism.

In an adult organism, most cells are differentiated and are in a quiescent state [44]. It is worth emphasizing that this state is characterized by a prolonged arrest of proliferation, but it is fundamentally different from CS [45]. First of all, the arrest of growth in this case is not a consequence of DNA damage. Secondly, this arrest can be reversed: with certain stimuli, differentiated cells in the G0 phase of the cell cycle can re-enter the cycle and start proliferating. One such stimuli is the disruption of the functioning of tissues or organs caused by damage. In this case, quiescent cells, such as skin fibroblasts, smooth muscle cells, endothelial cells, the epithelial cells of many internal organs, including the pancreas, liver, kidneys, lungs, prostate and mammary glands, may begin to proliferate to replace cells in damaged areas [44]. Most of these types of cells are susceptible to both replicative and premature senescence [40, 46-48]. It is interesting, however, that damage does not induce CS equally in all types of cells [49]. For example, the epithelium is a very dynamic tissue, characterized by a high rate of renewal. In this tissue homeostasis is supported mainly through the death of damaged and the proliferation of normal cells, and, therefore, epithelial cells are more prone to apoptosis than they are to triggering CS [50]. The opposite is typical for the stromal cells that form the framework of all internal organs. These cells are resistant to apoptosis and are more likely to enter the state of senescence [49].

Despite the examples of recovery of proliferation by certain types of epithelial and stromal cells described above, *in vivo *most of the cells that perform specialized functions are in the terminal differentiated state and, with rare exceptions, are incapable of proliferating even in the case of severe damage [44]. In this case, regeneration is carried out by the division and differentiation of adult stem cells (SCs). Pools of resident stem cells have been found in virtually every tissue [51]. However, it turns out that adult SCs are also susceptible to senescence. First of all, these cells have no active telomerase, and, therefore, SCs, just like all other proliferating cells, experience replicative senescence [52, 53]. Secondly, recently it has been demonstrated that various stress factors can induce premature senescence of SCs [54-56]. Taking into account the unique role of SCs in tissue regeneration in an adult organism, one has to emphasize the negative consequences of the aging of these cells. The senescent SCs lose their ability to proliferate, and their migration activity and differentiation potential decrease [57]. Thus, CS leads to a gradual depletion of the pool of functional SCs: on the one hand, their number decreases, and on the other, they cease to respond properly to external stimuli [58]. There is a view today that holds that SCs senescence is related to organismal aging, and the amount of data describing the contribution of senescent SCs to the development of various age-related diseases is increasing [58, 59].

While speaking about CS, one also has to mention a very special case: the senescence of transformed cells. Given that cancer cells possess unlimited proliferative potential, this, of course, is not about replicative, but about premature, senescence. In normal proliferating cells, premature CS is a physiological response to stress. However, in transformed cells, it can be induced only under specific circumstances, such as treatment with chemotherapeutic agents, irradiation, and overexpression of growth inhibitory genes [60]. Therefore, the induction of CS in transformed cells can be considered as one of the ways available to arrest tumor growth [60].

## “SOCIAL LIFE” OF SENESCENT CELLS

**Fig. 2:**
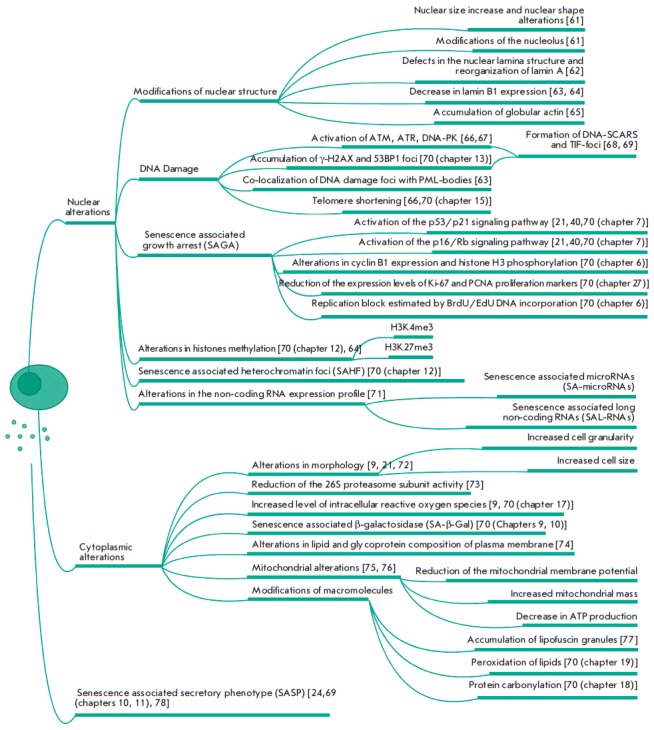
Biomarkers of senescent cells. The main features of senescent cells and references describing experimental approaches for their estimation are provided

It is well known that the main features of CS are similar across its different forms and different types of proliferating cells [40]. *Figure 2* shows the most important “individual” intracellular changes that accompany CS, which are subdivided into events occurring in the nucleus and in the cytoplasm. The change in the secretory profile occupies a special place among the modifications accompanying CS. It is generally accepted that the senescence- associated secretory phenotype (SASP) defines the engagement of senescent cells in a wide range of processes, such as reparation, propagation of senescence, immune clearance, embryogenesis, and tumorigenesis [29, 31, 38 , 79, 80].

**Classification of SASP factors**

The term SASP was first used in 2008 to refer to the factors secreted by senescent cells [24]. The following classification of SASP components has been adopted: soluble signaling factors, proteases, insoluble extracellular matrix proteins, and non-protein components [78]. SASP factors can be divided into the following groups based on molecular mechanisms [81]:

*1) Factors binding to a receptor. *This group includes soluble signaling molecules, such as cytokines, chemokines, and growth factors. These factors can influence cells of the microenvironment by interacting with the corresponding surface receptors on their membranes and, thus, triggering various intracellular signaling cascades [82, 83]. The most well known representatives of this group are interleukins IL-6, IL-8, IL-1a, chemokines GROα, GROβ, CCL-2, CCL-5, CCL-16, CCL-26, CCL-20, and the growth factors HGF, FGF, TGFβ, and GM-CSF.

*2) Factors acting directly. *This group includes matrix metalloproteases MMP-1, MMP-10, MMP-3 and serine proteases: the tissue plasminogen activator (tPA) and urokinase plasminogen activator (uPA). These factors are capable of cleaving membrane-bound proteins, destroying signaling molecules and remodeling the extracellular matrix, to enable senescent cells to modify their microenvironment [84]. Small non-protein components, such as reactive oxygen (ROS) and nitrogen species that damage neighboring cells, can also be included in this group [78, 85].

*3) Regulatory factors. *This group includes tissue inhibitors of metalloproteases (TIMP), the plasminogen activator inhibitor (PAI), and insulin-like growth factor binding proteins (IGFBP). These factors do not have their own enzymatic activity. However, when they bind to factors from the first and second groups, they regulate their functioning. For example, TIMP inhibits the activity of most MMPs [86], PAI-1 functions primarily as an inhibitor of tPA and uPA [87], and IGFBP function as IGF transport proteins [88].

In addition to all the factors mentioned above, which are secreted by senescent cells, another component has recently begun to be viewed as part of SASP: extracellular vesicles, in particular vesicles associated with microRNAs [89]. It turns out that such vesicles can affect neighboring cells and cells located at a considerable distance, both by initiating and suppressing CS, depending on the composition of microRNAs.

**Fig. 3:**
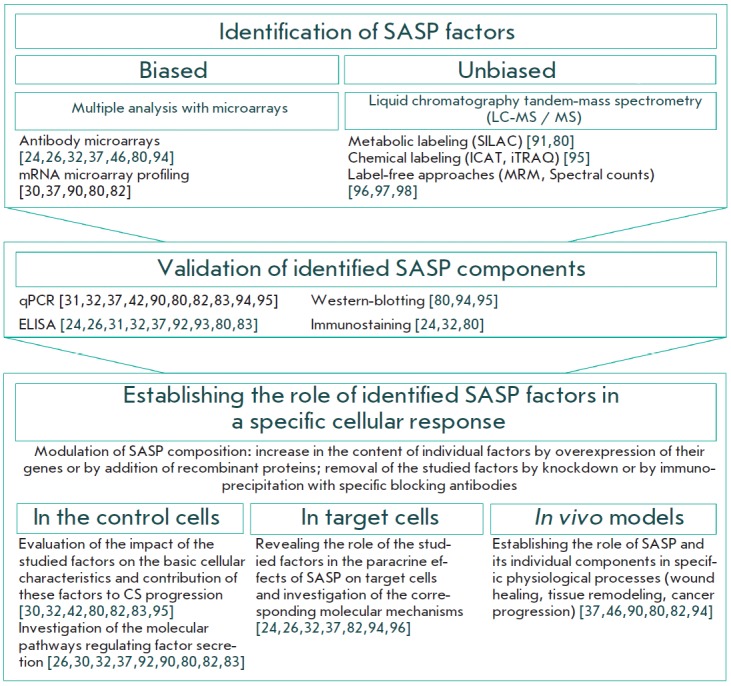
Experimental approaches to study SASP and to identify the functional role of its individual components

It should be emphasized that the specific qualitative and quantitative composition of the secreted factors largely depends on the type of cells and the inducer of senescence, which makes it very difficult to study this CS feature. Several approaches to the study of SASP and elucidation of the functions of its individual components have been described to date. The main approaches are presented at *Fig. 3*.

**Mechanisms of SASP regulation**

It is well known that cellular senescence is not a onetime phenomenon, but one that develops over time [99]. Remarkably, SASP has also recently begun to be viewed as a dynamic process which can be subdivided into several phases [16]. It is believed that the first phase of secretion begins immediately after DNA damage and lasts for the first 36 hours. It should be noted that the onset of this phase is not sufficient evidence in favor of initiation of senescence, since it does not preclude complete repair or apoptosis [99]. The next phase is the “early” SASP phase, which continues for several days after the initiation of CS. It is during this period that the most important SASP factors, for example IL-1α, start to appear. During the next 4–10 days, the secretion of most factors intensifies due to the autocrine effect of SASP, which ultimately leads to the formation of “mature” SASP [16]. Such a wave-like secretion of factors during the development of CS is largely attributed to positive feedback loops and complex regulatory mechanisms. The most common mechanisms for SASP regulation are presented below.

It should be noted that SASP is regulated both at the transcriptional and post-transcriptional levels. The key role in the regulation of SASP components expression, including IL-6, IL-8, CXCL1, and CXCR2, belongs to the nuclear factor kappa-light-chain-enhancer of activated B cells, NF-kB [100-102]. For most of these factors, control over transcription is achieved through positive feedback loops. A vivid example of such “self-amplifying” loops is the regulation of IL-1α secretion [15, 103]. It has been reported that another transcription factor, C/EBPβ, is also involved: by binding directly to the promoter of the *IL-6 *gene, where it initiates its expression [82, 104].

At the post-transcriptional level of SASP regulation, it is customary to identify DDR (DNA Damage Response)-dependent and independent mechanisms [15]. As mentioned above, one of the most important features of CS is the DNA damage response. It has been shown that knockdowns of such DDR components as ATM, Chk2, NBS1, and H2AX reduce the expression and, accordingly, the secretion of a number of SASP factors, including IL-6 and IL-8 [104-106]. Despite evidence that DDR is involved in SASP regulation, the detailed mechanisms for their relationships are not fully understood. The signaling pathways known today are associated with the ability of DDR components, in particular ATM kinase, to somehow regulate NFkB activity. For example, ATM can form complexes with the NEMO protein, which, due to the initiation of DDR, are exported from the nucleus to the cytoplasm, where NEMO binds to and activates IKK kinase. IKK promotes the dissociation of the inhibitory IkB protein from its complex with NF-kB and activation of the latter [107]. More recently, the involvement of the transcription factor GATA4 in the DDR-dependent mechanism of SASP regulation has been demonstrated [108]. Normally, GATA4 is degraded by p62-mediated autophagy. However, autophagy is suppressed in most senescent cells, and, therefore, GATA4 stabilizes, and this process is ATM-dependent. The accumulation of GATA4 in senescent cells facilitates the initiation and maintenance of NF-kB activity.

In the DDR-independent mechanism of SASP regulation, the key role is played by the stress-kinase p38, which is involved in the activation of the p16Ink4a/Rb signaling pathway that mediates the arrest of the cell cycle in senescent cells [109]. A number of studies have demonstrated that suppression of p38 expression prevents the secretion of most of the cytokines, chemokines and growth factors that make up SASP [110, 111]. In addition, maintaining p38 in the active state for a long time can initiate SASP in the absence of any other stimuli that cause senescence [110]. The following chain of signaling events was proposed for the mechanism of p38 involvement in SASP regulation: p38 activates its underlying targets – MSK1 and MSK2 kinases – which then phosphorylate p65, the transactivation subunit of NF-kB, thereby initiating the expression of many SASP factors [16, 112 , 113].

Recently, the role of the mTOR protein in the regulation of SASP was identified [114, 115]. On the one hand, it has been shown that mTOR can control the translation of IL-1α and thus regulate SASP [115]. On the other hand, mTOR controls the translation of MK-2 kinase, which phosphorylates the specific RNA-binding protein ZFP36L1, preventing the degradation of the transcripts of a large number of SASP factors [114]. Another possible option for mTOR involvement in the regulation of SASP is associated with the presence on the trans side of the Golgi apparatus of a special compartment (TOR-autophagy spatial coupling compartment, TASCC) in which autolysosomes and mTOR are accumulated during senescence [116]. It is assumed that the accumulation of mTOR in this compartment helps accelerate the synthesis of SASP factors.

The regulatory mechanisms described above are the most well studied to date. However, the huge diversity of the proteins included in SASP, as well as the fact that the composition of the secreted factors depends on the cellular context and the type of senescence, leads to an increase in the number of studies focused on detailing the molecular mechanisms of SASP regulation. In most publications, the emphasis is on the relationship between regulatory mechanisms and the functional role of SASP in specific biological processes, which will be discussed in the next chapter. It should be noted that most of the research is performed on cancer cells or on fibroblasts. Paradoxically, despite the obvious biological significance of stem cell senescence, the molecular mechanisms of SASP regulation in these cells are relatively poorly studied.

**Functional role of SASP**

To understand the mechanisms that mediate the involvement of SASP in a variety of biological processes, one first needs to answer a fundamental question: why do senescent cells secrete so many specific factors? Based on composition, it is logical to assume that* in vivo *SASP can serve as a signal that indicates the appearance of senescent cells in the body. Schematically, this process can be described as follows: the secreted proinflammatory cytokines and chemokines form the focus of the inflammation and attract cells of the immune system to the areas of senescent cells localization for their elimination; the proteins that remodel the extracellular matrix facilitate the entry of immune system cells to these areas; and the secreted growth factors stimulate the proliferation of neighboring cells for subsequent replacement of the removed cells. In a young healthy organism, this mechanism is well regulated. However, with age or in case of lesions, its effectiveness can be significantly impaired, leading to the accumulation of senescent cells in the population and, consequently, to prolonged secretion of SASP factors. Therefore, the outcome of the influence of SASP components on the microenvironment is defined by the balance between how long the senescent cells remain in the population and their rate of elimination by the cells of the immune system [12, 14-16]. Thus, the effects of SASP that are positive for the organism are due to the temporary presence of senescent cells, whereas its negative effects are associated with the accumulation of senescent cells and the emergence of a focus of chronic inflammation.

The opposite consequences of the phenomenon of “auto/paracrine senescence” can be cited as an example of such time dependence of the SASP effects. It is established that once the molecules secreted by the senescent cells get into the extracellular space, they are able to act on adjacent normal cells through the auto/ paracrine pathway and initiate the arrest of the cell cycle, stop proliferation, greatly accelerating the development of CS in the population [80, 83, 117]. For example, a conditioned medium derived from replicatively, oncogen or etoposide-aged fibroblasts containing high levels of IL-1, IL-6, and TGFβ contributes to an increase in the level of ROS, damage to DNA and, accordingly, the onset of senescence in normal cells [117]. The role of such SASP factors as activin A, GDF15, VEGF, CCL2, and CCL20 chemokines in the regulation of senescence has also been established [80]. It has been shown that compounds inhibiting the activity or the binding receptors of these factors prevent the development of senescence in a population of fibroblasts. According to our preliminary results, the cultivation of endometrial stem cells in a conditioned medium obtained from senescent cells also initiates premature senescence in young cells, with the PAI-1 protein playing an important role in this process. Returning to the duality of the cummulative effects of SASP, it should be noted that autocrine senescence plays a positive role in the case of temporary presence of senescent cells: first of all, it prevents the proliferation of the damaged cells, and secondly, it activates the immune response that leads to their removal [28-31, 118].

However, the accumulation of senescent cells and the prolonged secretion of SASP, which promotes the spread of premature senescence to neighboring cells, can lead to disruption in the functioning of tissues, accelerate the development of aging, and various age-associated diseases [33, 119]. For example, the increased secretion of matrix metalloproteases by senescent cells plays an important role in the progression of such pathologies as ischemic heart disease, osteoporosis, and osteoarthritis [120, 121]. Senescent smooth muscle cells secreting large amounts of pro-inflammatory cytokines are involved in the development of atherosclerosis [122]. The increased secretion of TNFα by senescent T cells is involved in the mechanism of bone loss [123]. It is also known that overexpression of IL-6 can lead to hyperinsulinemia, liver inflammation and pulmonary hypertension [124, 125]. In addition, the term ‘inflammaging’ has been introduced comparatively recently to describe the non-infectious chronic systemic inflammation that accompanies aging, and SASP factors secreted by old cells play a crucial role in its progression [34].

Another manifestation of the duality of the functional effects of SASP is its tumor-suppressing and tumor-promoting activities [2, 14, 28, 78]. A number of works that highlight the tumorigenic role of SASP have demonstrated that factors secreted by senescent fibroblasts stimulate the proliferation of various precancerous and transformed cell lines [24, 25, 126, 127]. Later, it was established that SASP induces an epithelial- mesenchymal transition and enhances the invasion of cells in the culture of precancerous epithelial cells, in particular through an increased content IL-6 and IL-8 [24]. It has been established that SASP factors secreted by senescent stem cells also contribute to the progression of cancer, accelerating the proliferation and migration of transformed cells [57]. For example, SASP factors secreted by SCs stimulate the division and migration of breast cancer cells both *in vitro *and in a mouse model [57]. In addition, it was established that senescent SCs secreting large amounts of IL-6 and IL-8 increase the resistance of breast cancer cells to cisplatin [26]. Based on the data available to date, it is most likely that SASP components induce proliferation, survival, and metastasis in already committed precancerous cells [14].

The tumor suppressing function is based on the ability of SASP factors to attract cells of the immune system to eliminate damaged senescent cells. Thus, a mouse model shows that Ras overexpression results in oncogen-induced hepatocyte senescence, which is accompanied by activation of SASP, stimulation of the CD4^+^-mediated immune response and, as a consequence, in the removal of these cells [28]. Another piece of evidence of the tumor-suppressing role of SASP was also obtained in a mouse model of hepatocarcinoma: however, in this case CS was induced by overexpression of p53 [29]. The secretion of various chemokines by senescent cancer cells led to the recruitment of natural killers (natural killers, NK) for their clearance. Remarkably, the removal of CCL2 chemokine by antibodies prevents the recruitment of NK cells and reduces the elimination of senescent cells.

The involvement of SASP in the regeneration of tissues deserves special attention. It is known that SASP factors can influence the signaling and differentiation of stem cells [33, 128, 129]. For example one of the key components of SASP, IL-6, promotes the induction and maintenance of pluripotency, in particular by regulating the expression of Nanog [130, 131]. Moreover, *in vivo *experiments have shown that secretion of SASP promotes the reprogramming of microenvironment cells [32]. This SASP-mediated tissue regeneration is another example of the time dependence of the cummulative effects of SASP. In a young organism, shortterm action of SASP promotes tissue regeneration through temporary reprogramming and subsequent proliferation and differentiation of neighboring cells, whereas in an elderly organism ineffective elimination of senescent cells and prolonged secretion of SASP can lead to a prolongation of the dedifferentiated state of neighboring cells, and, accordingly, to the inhibition of regeneration [33].

Interesting results concerning the role of SASP in tissue regeneration and remodeling were obtained by studying the molecular mechanisms of wound healing. It has been established that senescent fibroblasts and endothelial cells can be detected at wound sites for several days, which promote wound healing through secretion of PDGF-A, the SASP factor responsible for the differentiation of myofibroblasts [31]. In addition, the role of SASP in tissue remodeling during embryonic development has been established [31, 37-39]. It has been shown that SASP-mediated remodeling occurs both from the maternal body and from the embryo. For example, SASP was implicated in the remodeling of the maternal vasculature in early pregnancy [131]. Senescent cells appear in the process of embryonic development and use SASP to act as a primary signal that triggers macrophage-mediated cell removal, which is necessary for the proper development of individual embryonic structures [31, 38, 39].

## CONCLUSION

Summing up all the above, let’s revisit the last turn of the spiral, which corresponds to the current stage in the history of cellular senescence studies, and once again emphasize the pleiotropy of CS effects. It is obvious that the experimental approaches that involve the elimination of senescent cells from the body and considered as “anti-aging” therapy can have a number of concomitant, undesirable consequences. Therefore, the most promising approach seems to be the development of strategies aimed at modulating the composition of the factors secreted by old cells, in order to enhance the positive and minimize the potential negative effects of SASP. Modulation of the SASP factors of senescent SC acquires particular importance in this context. Taking into account that at present the most probable mechanism of SC influence on tissue repair is their paracrine activity, the issue of changes in the secretory profile of SC as a result of their aging becomes very urgent and requires additional studies.
